# Genome-wide identification and characterization of DTX family genes highlighting their locations, functions, and regulatory factors in banana (*Musa acuminata*)

**DOI:** 10.1371/journal.pone.0303065

**Published:** 2024-06-06

**Authors:** Al Amin, Md. Darun Naim, Nurul Islam, Md. Nurul Haque Mollah

**Affiliations:** 1 Department of Statistics, Bioinformatics Laboratory, Faculty of Science, University of Rajshahi, Rajshahi, Bangladesh; 2 Department of Zoology, Faculty of Biological Sciences, University of Rajshahi, Rajshahi, Bangladesh; 3 Department of Botany, Faculty of Biological Sciences, University of Rajshahi, Rajshahi, Bangladesh; Nuclear Science and Technology Research Institute, ISLAMIC REPUBLIC OF IRAN

## Abstract

The detoxification efflux carriers (DTX) are a significant group of multidrug efflux transporter family members that play diverse functions in all kingdoms of living organisms. However, genome-wide identification and characterization of DTX family transporters have not yet been performed in banana, despite its importance as an economic fruit plant. Therefore, a detailed genome-wide analysis of DTX family transporters in banana (*Musa acuminata*) was conducted using integrated bioinformatics and systems biology approaches. In this study, a total of 37 DTX transporters were identified in the banana genome and divided into four groups (I, II, III, and IV) based on phylogenetic analysis. The gene structures, as well as their proteins’ domains and motifs, were found to be significantly conserved. Gene ontology (GO) annotation revealed that the predicted DTX genes might play a vital role in protecting cells and membrane-bound organelles through detoxification mechanisms and the removal of drug molecules from banana cells. Gene regulatory analyses identified key transcription factors (TFs), *cis*-acting elements, and post-transcriptional regulators (miRNAs) of DTX genes, suggesting their potential roles in banana. Furthermore, the changes in gene expression levels due to pathogenic infections and non-living factor indicate that banana DTX genes play a role in responses to both biotic and abiotic stresses. The results of this study could serve as valuable tools to improve banana quality by protecting them from a range of environmental stresses.

## 1. Introduction

Plants regularly encounter toxins derived from external sources such as organisms and pathogenic microorganisms, as well as toxins generated from their internal metabolic processes. The processes of detoxification and disposal of harmful substances, both exogenous and endogenous, play vital roles in the survival and growth of plants. There are numerous potential mechanisms for discarding and detoxification of toxic compounds, for example, sequestration in the vacuole [1], alteration by enzymes endogenously [2], extrusion from the cells [3], reducing the entry by decreasing the permeability of cell membrane [3], and also by ribosome modification [4]. The multidrug and toxic compound extrusion (MATE) proteins extrude structurally and chemically dissimilar substrates out of cells [5, 6]. It is also known as detoxification efflux carrier (simply DTX), since it plays a vital role in cell detoxification. The DTX proteins are a sub-family of the multidrug-resistant transporter (MDRT) family. There are five kinds of MDRT proteins including ATP-binding cassette superfamily (ABC) also referred to as traffic ATPases, DTX, small gene multidrug resistance family (SMR), major facilitator superfamily (MFS) and resistance-nodulation-division family (RND) [3, 7]. The ABC family members are considered the primary transporters that are energized by ATP [3]. The transmembrane transport mechanism of the ABC system is driven by energy release, such as through methyl transfer reactions, electron transport, and ATP hydrolysis [8]. However, the members of DTX, SMR, MFS, and RND families are secondary transporters. Secondary transport proteins utilize the electrochemical potential generated by the concentration disparity of substances across the membrane to drive substrate transport [9]. The DTX proteins are widely present in all kingdoms of living organisms [10]. These proteins consist of 400–700 amino acids and possess 8–12 transmembrane domains [11, 12].

Diener et al. (2001) first isolated AtALF5, a DTX transporter in *Arabidopsis*, and associated it with the development of epidermal cells and exportation of toxic compounds in roots [13]. Several studies have been carried out in recent years to characterize the function of DTX proteins in the model plant *Arabidopsis thaliana*. These studies have demonstrated that DTX proteins have a variety of roles. One example of a DTX-type transporter is encoded by the *Arabidopsis* transparent testa 12 (tt12) gene. This transporter acts as a vacuolar flavonoid/H+ antiporter in proanthocyanidin-accumulating cells of the seed coat. In *Medicago truncatula* and *Arabidopsis*, it plays a role in the vacuolar uptake of epicatechin 3’-O-glucoside, a key component of proanthocyanidin biosynthesis [14]. The *A*. *thaliana* DTX1 (AtDTX1) is a transporter protein which is located in the plasma membrane, contributing to the efflux of TEA and berberine toxic compounds from the cytoplasm [11]. The homologous DTX genes HvAACT1 (aluminum [Al]-activated citrate transporter 1) and OsFRDL1 work as a citrate transporter required for efficient translocation of iron (Fe) in barley (*Hordeum vulgare*) and rice (*Oryza sativa*), respectively [15].

AtDTX18 increases plant defense mechanisms against the fungal pathogen, *Phytophthora infestans* [16]. A DTX protein is encoded by Arabidopsis Activated Disease Susceptibility 1 (ADS1) which is involved in plant disease resistance [17]. A cotton DTX gene enhances abiotic tolerance in genetically-modified arabidopsis [18]. Thus, DTX family genes may play a vital role in plant growth and development, plant resistance to biotic and abiotic stress and diseases, leading to better quality plant productions. Several studies on DTX family genes have identified 56 genes in *Arabidopsis* [11], 48 in potato [19], 49 in maize [20], 45 in rice [21], 117 in soybean [22], and 66 in apple [23].

Banana (*Musa acuminata*), a monocotyledonous perennial of significant agricultural importance, is extensively cultivated throughout tropical and subtropical nations as a crucial fruit crop [24, 25]. It ranks fourth among the most crucial staple crops globally, relied upon by over 400 million people for both food security and income [26, 27].

**Fig 1:**
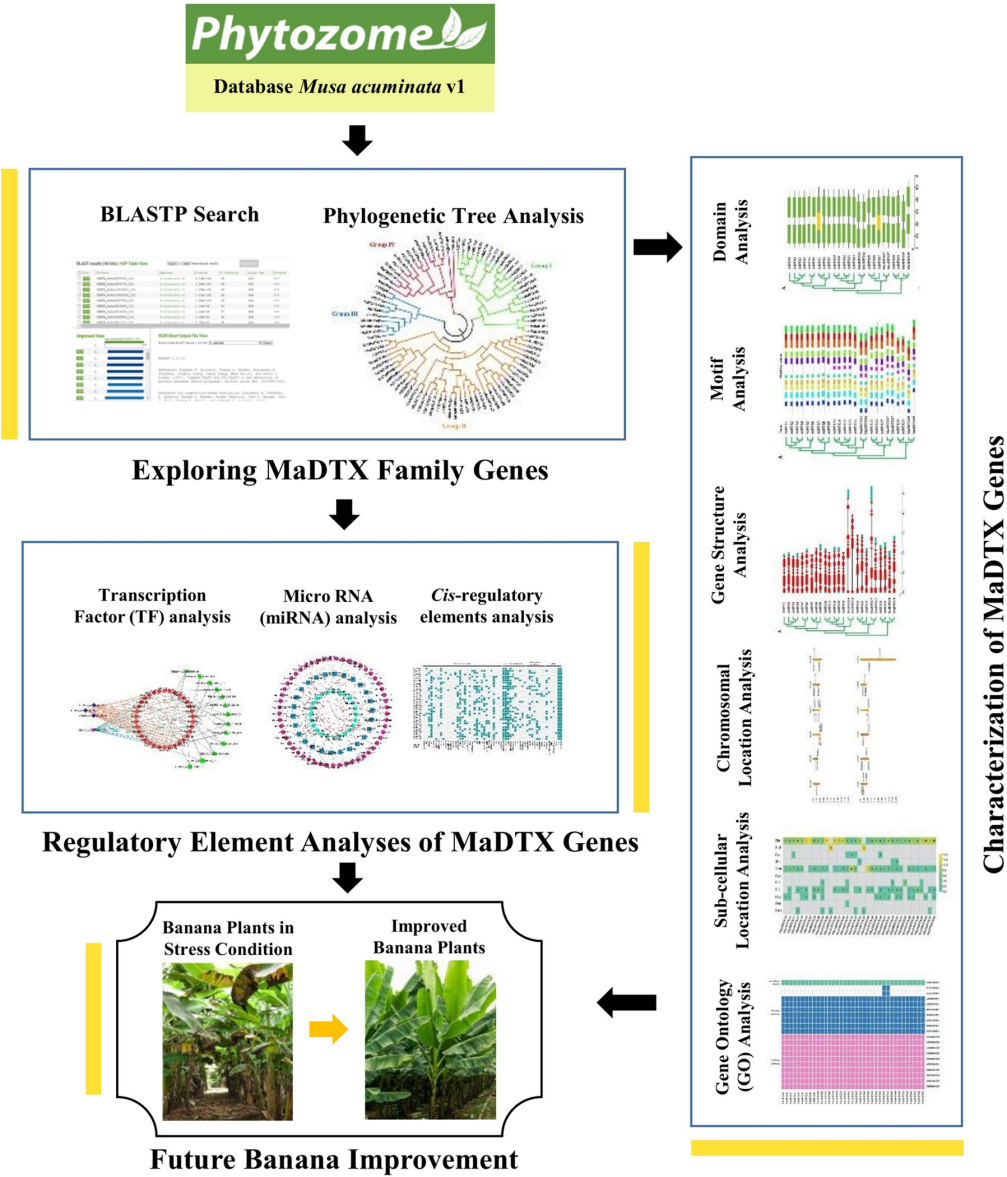
The procedural diagram outlining the operations conducted in this study.

It boasts a diverse array of vitamins, antioxidants, minerals, dietary fiber, starch, saccharides, and cellulose, constituting its nutritional richness [28]. The antioxidant properties of banana play a vital role in the prevention of a number of ailments. These includes hypertension, cancer, diabetes, cardiovascular disease, diarrhea, and infectious diseases [29]. Plant breeders are, therefore, very interested in several agronomic characteristics of banana, such as its resistance to biotic and abiotic stress, disease, and pests, for the production of higher-quality fruits. In this case, the DTX family genes may play a vital role similar to other plants as discussed earlier. However, so far, no study has yet been conducted on DTX family genes in banana. The discovery of DTX family genes through wet-lab experiment is time consuming, laborious and expensive. Computational approaches reduce the volume of works in this regard. Therefore, in this study, *in-silico* analysis for genome-wide identification and characterization of DTX family genes was considered. The pipeline of this study is provided in Fig 1.

## 2. Materials and methods

### 2.1 The data source and descriptions

To explore MaDTX genes from banana (*Musa acuminata*) genome, its genome and proteome sequences were retrieved from the Phytozome database (genome ID: 304, NCBI taxonomy ID: 4641, https://phytozome-next.jgi.doe.gov/info/Macuminata_v1) [30, 31]. The banana genome has been previously used to target genes and transcription factors for translational research, as well as for the genome wide identification of RNA interference (RNAi) genes [32, 33]. In this study, the genome dataset was used to investigate MaDTX genes through a BLASTP search against the *A*. *thaliana* DTX (AtDTX) genes. Additionally, 56 DTX transporters from *Arabidopsis*, as previously documented, were downloaded from The *Arabidopsis* Information Resource (TAIR) database (https://www.Arabidopsis.org/) [11] and genomic data of rice were downloaded from the Ensemble database (http://asia.ensembl.org/index.html) [34].

### 2.2 Integrated bioinformatics analyses

The integrated bioinformatics studies included BLASTP search, multiple sequence alignment (MSA), phylogenetic tree construction, functional domain analysis, exon-intron structure analysis, subcellular location detection, GO-terms identification, and regulatory factors analysis see Fig 1.

#### 2.2.1 Exploring DTX family genes

*2*.*2*.*1*.*1*. *BLASTP search with respect to AtDTX proteins*. Banana putative DTX protein sequences were selected from its proteome at Phytozome database (v13; http://www.phytozome.net/) [31] through the Basic Local Alignment Search Tool (BLASTP) searches by using 56 *A*. *thaliana* DTX protein sequences as queries. Sequences were selected based on alignment scores of 50 or higher using the BLOSUM62 matrix, identity percentages of 45% or higher, coverage percentages of 70% or higher, and E-values of 10E-10 or lower. To retrieve the identified genes and proteins sequences, only the primary sequences were considered to avoid the redundancy. Information on selected genes/proteins, including their primary sequences, genomic length, protein length, chromosomal position, and the length of the open reading frame (ORF) was collected from the *M*. *acuminata* genome in the Phytozome database. Physical parameters such as molecular weight (MW) and theoretical isoelectric point (pI) were calculated using ProtParam (https://web.expasy.org/protparam/) [35].

*2*.*2*.*1*.*2*. *Phylogenetic analysis*.To denote the names of newly identified MaDTXs genes in *M*. *acuminata* genome with respect to the nomenclature of AtDTXs, a combined phylogenetic tree based on their protein sequences was constructed. To construct the phylogenetic tree, combined multiple sequence alignments (MSA) including MaDTXs, AtDTXs and OsDTXs protein sequences was performed by using the ClustalW algorithm [36] in MEGA11 software with default parameters [37]. The outcomes from the alignment were then utilized to generate a phylogenetic tree using the neighbor-joining (NJ) approach [38] in MEGA 11. The setup included the Poisson model with uniform rates among sites, using complete deletion for gaps and missing data. One thousand bootstrap replications were performed on the NJ tree [38].

#### 2.2.2 Characterization of MaDTX genes

*2*.*2*.*2*.*1*. *Conserved domain and motif analysis*. To characterize the conserved domains of DTX gene families in *M*. *acuminata*, the protein sequences were retrieved and subjected to analysis using the Protein Family (Pfam) database (https://www.ebi.ac.uk/Tools/hmmer/search/hmmscan) [39]. The analysis selected the maximum number of significant functional conserved domains of *M*. *acuminata* (MaDTX) proteins that are similar to those in *A*. *thaliana* AtDTX proteins. Pfam result was displayed using the TBtools, a Toolkit for Biologists integrating various biological data-handling tools [40]. To identify potential regulatory motifs, the MEME Suite (v. 5.4.1, https://meme-suite.org/meme/tools/meme) was utilized with the following parameters: zero or one occurrence per sequence for site distribution, motif width ranging from 6 to 50 nucleotides, and a maximum of 12 motifs [41].

*2*.*2*.*2*.*2*. *Gene structure and chromosomal localization analysis*. The online Gene Structure Display Server (http://gsds.gao-lab.org/) [42] was utilized to generate maps of exon-intron structure by aligning coding sequences (CDS) of AtDTX and MaDTX to their corresponding genomic sequences. The chromosomal locations of predicted genes were mapped using the online tool MapGene2Chromosome V2.0 (http://mg2c.iask.in/mg2c_v2.0/) [43].

*2*.*2*.*2*.*3*. *Gene ontology and sub-cellular localization analysis*. The analysis of Gene Ontology (GO) was performed using the online tool available on the Plant Transcription Factor Database, (PlantTFDB, http://planttfdb.cbi.pku.edu.cn//) [44]. Genes were categorized across three distinct GO classifications: molecular function (MF), biological processes (BP), and cellular components (CC). Statistical significance was determined using Fisher’s exact test, and *p*-values were subsequently adjusted using Benjamini-Hochberg corrections. A *p*-value of less than 0.05 was considered statistically significant in evaluating the GO enrichment outcomes associated with the predicted genes. The WoLF PSORT web-based platform was used to predict the subcellular locations of MaDTX proteins within various cell organelles, with their protein sequences as input (https://wolfpsort.hgc.jp/) [45].

#### 2.2.3 Regulatory element analyses

*2*.*2*.*3*.*1*. *MaDTXs regulatory network analysis with trans-acting factors*. In this study, a comprehensive database of transcription factors in plants, PlantTFDB, (http://planttfdb.cbi.pku.edu.cn//) [44] was used to conduct the analysis of the predicted transcription factor families and their potential impact on gene regulation of DTX related genes in *M*. *acuminata*. Firstly, the transcription factors (TFs)/*trans*-acting factors were identified that were closely associated with DTX-related genes in *M*. *acuminata*. Subsequently, a regulatory network was established and visualized using Cytoscape 3.7.1 to identify key transcription factors and their interactions, providing a comprehensive understanding of the regulatory mechanisms underlying DTX-related gene expression [46].

*2*.*2*.*3*.*2*. *MaDTXs regulatory network analysis with microRNAs*. The extensively used Plant miRNA ENcyclopedia (PmiREN) was employed to retrieve micro-RNA (miRNA) datasets in *M*. *acuminata*. Then, to identify miRNAs targeting the banana MaDTX genes, the CDS sequences of putative MaDTX genes were searched for sequences complementary to miRNAs, using psRNATarget (https://www.zhaolab.org/psRNATarget/) [47] with default parameters.

*2*.*2*.*3*.*3*. *Prediction of cis-regulatory elements in the MaDTXs promoter regions*. To investigate the *cis*-acting regulatory elements in the promoter region of MaDTX family members, the 1.5kb upstream region of MaDTX genes extracted and uploaded to PlantCARE (http://bioinformatics.psb.ugent.be/webtools/plantcare/html/) database [48]. The collection of *cis*-regulatory elements was then divided into five categories: abiotic and biotic stress response, phytohormone response, plant growth and development, other functions, and unknown function. The identified promoter *cis*-acting regulatory elements of MaDTX are presented independently.

#### 2.2.4 MaDTXs gene expression pattern analysis

The gene expression profiles datasets with accession numbers GSE48563 and GSE134166 were downloaded from the National Center of Biotechnology Information (NCBI) Gene Expression Omnibus (GEO) database (https://www.ncbi.nlm.nih.gov/geo/) [49]. The datasets were used to extract TPM values (GSE48563) and FPKM (GSE134166) values for creating heatmaps with TBtools [40].

## 3. Results

### 3.1 Exploring DTX family genes

#### 3.1.1. Identification of DTX family genes in Banana genome

**Fig 2:**
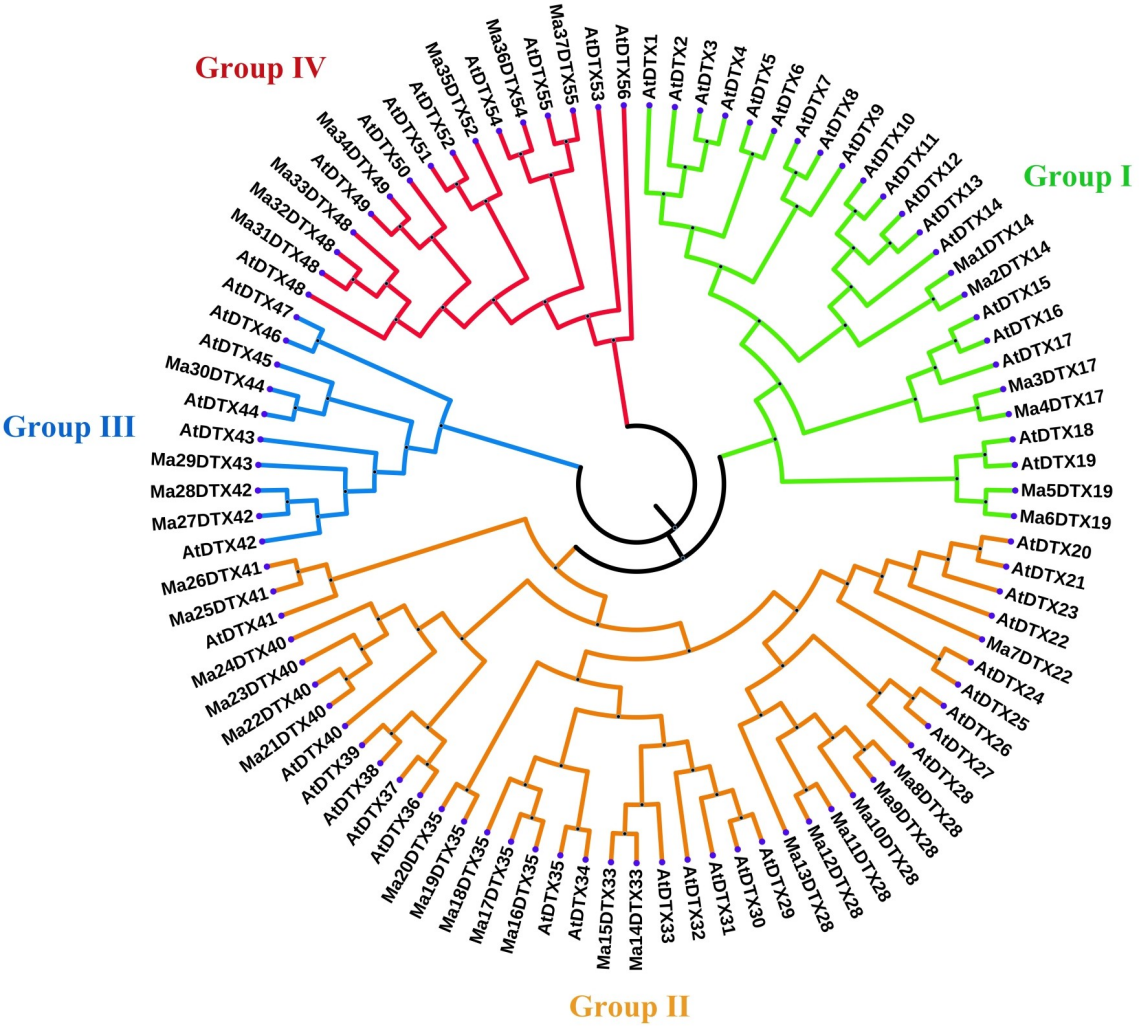
The neighbor-joining (NJ) phylogenetic tree of banana and *Arabidopsis* DTX proteins. Different groups are indicated by the different colors (Group I in green, Group II in orange, Group III in blue and Group IV in red). ‘MaDTX’ denotes DTX members from banana, ‘AtDTX’ denotes DTX members from *Arabidopsis*.

The BLASTP search identified a total of 37 MaDTX genes in the *M*. *acuminata* genome [24] with respect to the 56 AtDTX genes. To find the similarities among MaDTX and AtDTX genes, a neighbor-joining (NJ) phylogenetic tree was constructed (Fig 2) based on both MaDTX and AtDTX gene sequences S1 File. In accordance with the phylogenetic tree, the 37 MaDTX transporters divided into four cohorts, named as Groups (I, II, III, IV) corresponding to what was stated for *Arabidopsis* DTX [11]. The banana DTXs were given names in accordance with their grouping with known *Arabidopsis* DTX transporters. For example, in Ma*j*DTX14, Ma indicates *Musa acuminata*, *j* indecates *j*th MaDTX gene (*j* = 1,2,…37) and the last number 14 indicates the 14^th^ query AtDTX gene. Additionally, using the same method and parameters, the phylogenetic tree was constructed among the DTX family genes of *M*. *acuminate*, *Arabidopsis* and *O*. *Sativa* to find the similarities and dissimilarities among their DTX family genes S1 Fig and the phylogenetic tree showing similar clustering patterns. Each MaDTX group size was uneven, ranged from 4 to 20, and Group I being the largest and Group II the smallest. The number of MaDTX transporters in Groups I, II, III, and IV is 6, 20, 4, and 7, respectively (Fig 2).

The Table 1 lists the physiological and biochemical characteristics of every MaDTX protein, including each protein’s chromosomal location, molecular weight, isoelectric point (pI), gene length, and length of the ORF. The genomic length of the anticipated 37 MaDTX genes varied from 1401 bp to 7753 bp, and ORF ranged between 1197 bp and 1839 bp. The length of their translated MaDTX proteins ranges from 398 to 612 amino acids, which is close to DTX proteins found in *Arabidopsis* (400–700 amino acids) [11], but distinct from that found in soybean (80–593 amino acids) [22] and Populus (120–608 amino acids) [50]. The predicted molecular weights and the theoretical isoelectric point (pI) values of banana DTX proteins range from 43.03 to 64.93 kDa and 4.89 to 9.42, respectively.

**Table 1:** Basic information of predicted DTX gene family of banana.

Serial Number	Gene Name	Accession Number	Chromosomal Location	Gene Length (bp)	ORF length (bp)	No. of Intron	Molecular Weight (kDa)	Protein Length (aa)	PI
1	Ma1DTX14	GSMUA_Achr3T29080_001	chr3:28377816..28383043	5227	1332	6	48.46	443	8.56
2	Ma2DTX14	GSMUA_Achr4T03730_001	chr4:2969220..2973184	3964	1332	6	48.38	443	8.39
3	Ma3DTX17	GSMUA_Achr10T25360_001	chr10:29420358..29425752	5394	1521	7	55.18	506	5.53
4	Ma4DTX17	GSMUA_Achr11T13300_001	chr11:13479340..13482151	2811	1467	7	52.29	488	8.23
5	Ma5DTX19	GSMUA_Achr2T13840_001	chr2:15821067..15823212	2145	1320	6	47.85	439	6.3
6	Ma6DTX19	GSMUA_Achr4T27830_001	chr4:26505368..26508258	2890	1677	7	60.75	558	8.6
7	Ma7DTX22	GSMUA_Achr7T21610_001	chr7:24356037..24360794	4757	1458	7	53.20	485	6.53
8	Ma8DTX28	GSMUA_Achr3T21260_001	chr3:22236846..22242356	5510	1383	8	49.96	460	6.45
9	Ma9DTX28	GSMUA_Achr8T18130_001	chr8:21255624..21257815	2191	1467	7	53.26	488	9.24
10	Ma10DTX28	GSMUA_Achr7T11770_001	chr7:9362424..9366500	4076	1290	7	46.71	429	6.51
11	Ma11DTX28	GSMUA_Achr6T04190_001	chr6:2868816..2872202	3386	1299	9	47.17	432	5.9
12	Ma12DTX28	GSMUA_Achr7T12820_001	chr7:10315416..10321720	6304	1302	8	47.48	433	7.49
13	Ma13DTX28	GSMUA_Achr10T24960_001	chr10:29150449..29153453	3004	1446	7	52.76	481	8.57
14	Ma14DTX33	GSMUA_Achr2T03560_001	chr2:9228835..9234025	5190	1503	7	54.90	500	7.94
15	Ma15DTX33	GSMUA_Achr4T05060_001	chr4:3942662..3949184	6522	1539	8	56.36	512	5.47
16	Ma16DTX35	GSMUA_Achr1T15340_001	chr1:11541597..11545028	3431	1428	7	52.00	475	5.97
17	Ma17DTX35	GSMUA_AchrUn_randomT07700_001	chrUn_random:32622450..32624575	2125	1521	6	55.00	506	4.89
18	Ma18DTX35	GSMUA_Achr7T04510_001	chr7:3409042..3411536	2494	1197	7	43.03	398	5.58
19	Ma19DTX35	GSMUA_Achr5T01870_001	chr5:1119189..1121309	2120	1419	7	50.95	472	9.12
20	Ma20DTX35	GSMUA_Achr9T26370_001	chr9:30820718..30822705	1987	1401	7	51.11	466	8.97
21	Ma21DTX40	GSMUA_Achr2T14000_001	chr2:15919072..15923747	4675	1566	6	56.26	521	5.51
22	Ma22DTX40	GSMUA_Achr6T00360_001	chr6:260374..263663	3289	1224	7	43.72	407	8.1
23	Ma23DTX40	GSMUA_Achr4T23760_001	chr4:23760218..23762765	2547	1383	8	48.65	460	6.59
24	Ma24DTX40	GSMUA_Achr9T11610_001	chr9:7547851..7551031	3180	1488	6	53.65	495	7.03
25	Ma25DTX41	GSMUA_Achr4T22990_001	chr4:23213617..23216154	2537	1512	7	55.05	503	7.57
26	Ma26DTX41	GSMUA_Achr8T03000_001	chr8:2068939..2071863	2924	1497	7	54.27	498	8.8
27	Ma27DTX42	GSMUA_Achr3T29780_001	chr3:28791693..28798345	6652	1557	12	55.78	518	6.89
28	Ma28DTX42	GSMUA_Achr4T06960_001	chr4:5196322..5204075	7753	1509	12	53.96	502	6.31
29	Ma29DTX43	GSMUA_Achr8T31410_001	chr8:33081764..33085884	4120	1482	12	52.79	493	9.42
30	Ma30DTX44	GSMUA_Achr11T19700_001	chr11:20788502..20794794	6292	1557	12	55.30	518	9.11
31	Ma31DTX48	GSMUA_Achr5T08950_001	chr5:6516147..6517826	1679	1512	2	54.19	503	7.5
32	Ma32DTX48	GSMUA_Achr6T24500_001	chr6:25210532..25212260	1728	1608	1	57.60	535	6.45
33	Ma33DTX48	GSMUA_AchrUn_randomT15450_001	chrUn_random:73097247..73102736	5489	1692	4	60.33	563	8.93
34	Ma34DTX49	GSMUA_Achr5T20120_001	chr5:21819088..21820489	1401	1335	1	47.34	444	8.01
35	Ma35DTX52	GSMUA_Achr10T14020_001	chr10:22415572..22417597	2025	1839	2	64.93	612	8.83
36	Ma36DTX54	GSMUA_Achr11T09040_001	chr11:6974869..6976829	1960	1308	2	45.72	435	8.68
37	Ma37DTX55	GSMUA_Achr3T24100_001	chr3:24743247..24745048	1801	1290	1	44.91	429	8.81

### 3.2 Characterization of MaDTX genes of banana

#### 3.2.1. Conserved domain and motif analysis

**Fig 3:**
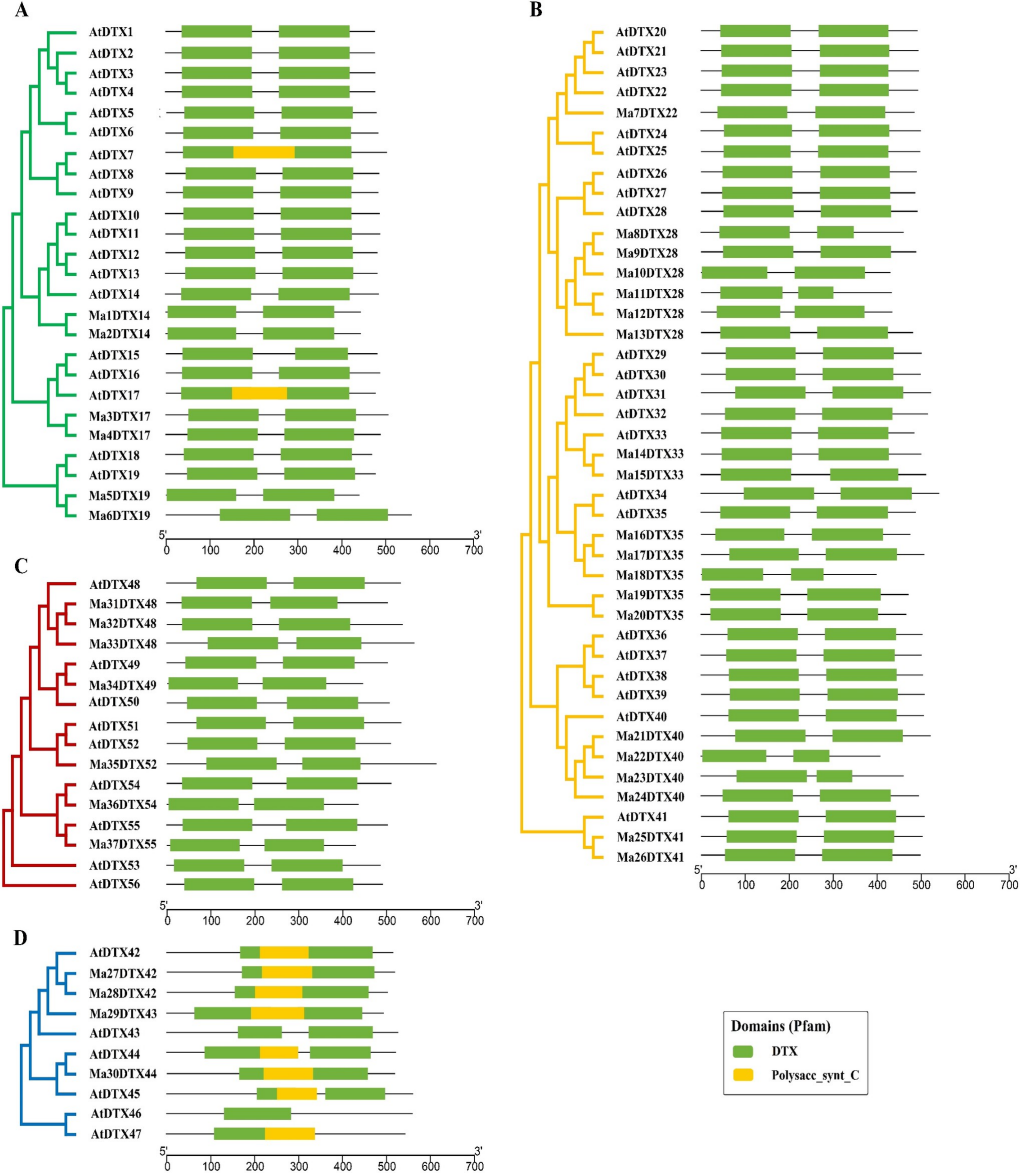
The conserved domains of the DTX family proteins in *M*. *acuminata* and *A*. *thaliana* were drawn using Pfam database information. (A) Group I, (B) Group II, (C) Group IV, (D) Group III.

**Fig 4:**
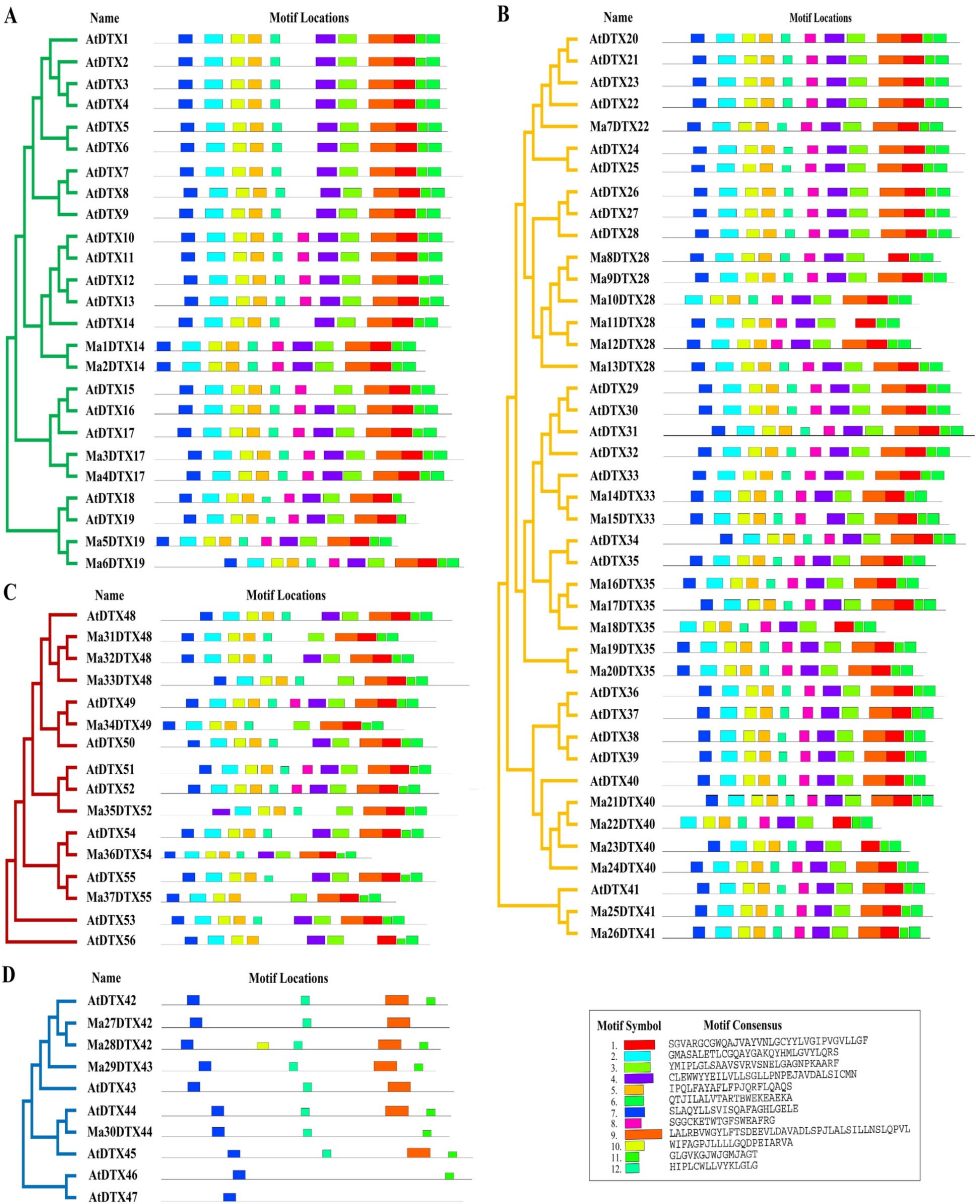
The conserved motif composition of the DTX family proteins in *M*. *acuminata* and *A*. *thaliana*. Different colors indicated different motifs, where top 12 motifs are displayed. (A) Group I, (B) Group II, (C) Group IV, (D) Group III.

The domains of the banana DTX family transporters are highly conserved, much like those of *Arabidopsis*, according to the findings of the functionally conserved domain analysis (Fig 3). The domain organization of MaDTXs and AtDTXs within the Group II & VI is conserved, consisting only of the DTX domains (Pfam: PF01554). In Group I, all the putative DTX protein sequences have DTX domains (Pfam: PF01554) except AtDTX7 and AtDTX17 which also contain Polysacc_synt_C domain (Pfam: PF14667.9). On the other hand, in Group III, the protein sequences have DTX and Polysacc_synt_C domains except the AtDTX43 and AtDTX46, which have only DTX domains. Using the Multiple Expectation Maximization for Motif Elicitation (MEME) program, the motifs of 93 DTX family members-37 banana DTX (new) and 56 *Arabidopsis* DTX-that encode proteins were examined (Fig 4). Twelve unique conserved regulatory motifs, referred to as motifs 1–12, were found. The majority of MaDTXs and AtDTXs proteins in the same group have similar motifs. There are nine to twelve motifs in groups I, II, and IV, and one to five motifs in group III. Twelve motifs were found in Ma3DTX17, Ma4DTX17, Ma5DTX19 and Ma6DTX19, which showed higher conservation with their paralogs AtDTX17 and AtDTX19. On the other hand, the Ma1DTX14 and Ma2DTX14 had 12 motifs but their paralog AtDTX14 had 11 motifs. In case of Group II, all proteins showed 12 motifs except Ma10DTX28, Ma18DTX35 and Ma22DTX40 which had 11 motifs and inconsistent with their paralogs, AtDTX28, AtDTX35 and AtDTX40.

#### 3.2.2. MaDTXs gene structures analysis

**Fig 5:**
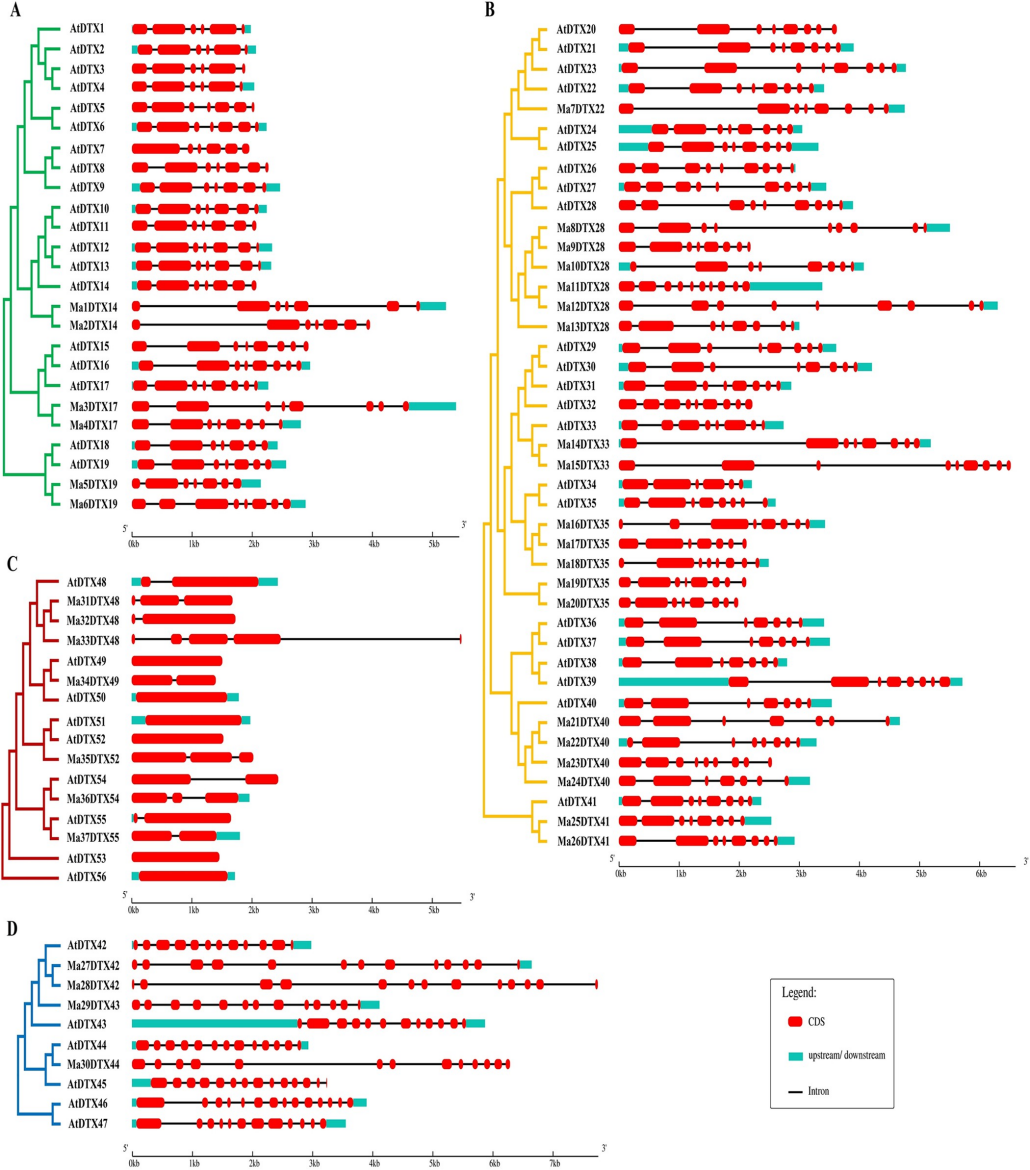
Exon-intron structure of the DTX family genes in *M*. *acuminata* and *A*. *thaliana*. The rounded rectangle boxes represent CDS, lines represent introns. The scale at the bottom can be used to estimate the length of genes. (A) Group I, (B) Group II, (C) Group IV, (D) Group III.

MaDTXs gene structure analysis was performed to offer additional understanding of the diversity of DTX gene structures in banana using a comparative analysis between the predicted coding sequences (CDSs) and the corresponding genomic sequences. The patterns of exons and introns within the predicted genes were more similar to those found in DTX genes from the model plant *A*. *thaliana* across different groups of the phylogenetic tree, as was expected (Fig 5). The MaDTX genes displayed diversity in the count of introns, ranging from 1 to 12, exhibiting substantial differences among distinct groups. The MaDTX genes within group III exhibited the greatest intron disorder, characterized by the presence of 12 disrupted introns. The MaDTX genes in the Group II, the second largest group in terms of intron disruption with 6–9 introns. All MaDTX genes of Group IV with 1–4 introns whereas the DTX genes in Group I has 6–7 numbers of intron disruption.

#### 3.2.3. Chromosomal location analysis of MaDTXs

The MaDTX genes were distributed unevenly on the eleven distinct chromosomes, including one unknown chromosome, of the entire banana genome S2 Fig, Table 1. MaDTX genes are distributed among chromosomes 1 to 11, with chromosome 4 having the greatest number of MaDTX genes and chromosome 1 having just one gene. Chromosome 2, 5, 6, 8, 10 and 11 contain 3 MaDTX genes, whereas chromosome 3 and 4 contain 4 genes. The unknown chromosomes and 9 contain similar number of genes.

#### 3.2.4. The sub-cellular localization of the MaDTX proteins

**Fig 6:**
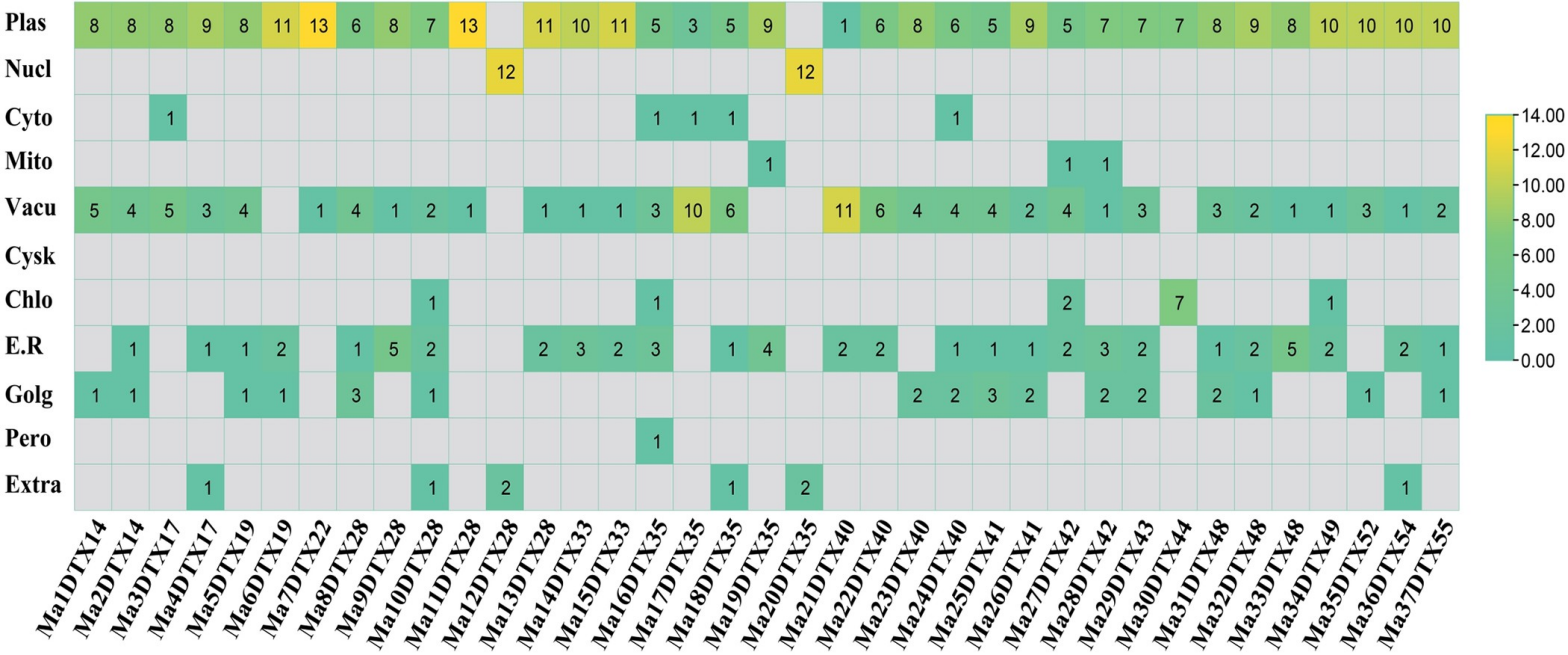
Sub-cellular localization of the predicted MaDTX proteins in *M*. *acuminata*. The numbers roughly indicate the number of nearest neighbors to the query predicted protein sequences which localize to each site. Here, plasma membrane (Plas), nucleus (Nucl), cytoplasm (Cyto), mitochondria (Mito), vacuole (Vacu), cytoskeleton (Cysk), chloroplast (Chlo), endoplasmic reticulum (E.R), golgi apparatus (Golg), peroxisome (Pero), and extracellular (Extra). The numbers roughly indicate the number of nearest neighbors to the query predicted protein sequences which localize to each site.

The location of specific proteins within a eukaryotic cell is associated with the cell’s biological functions. The Sub-cellular localization of MaDTX Proteins help to comprehend of their cellular functions. Most MaDTX proteins were expected to be primarily located in the plasma membrane, with subsequent localization in the vacuole, endoplasmic reticulum, and the golgi bodies (Fig 6). A small number of predicted protein was found to be present in chloroplast and cytoplasm. Only two predicted proteins, Ma12DTX28 and Ma20DTX35 were present in nucleus. The significant presence of proteins encoding DTX genes in the plasma membrane indicates their main role in preserving the integrity of the membrane by expelling various substances from the plants.

#### 3.2.5. MaDTXs gene-set enrichment analysis

**Fig 7:**
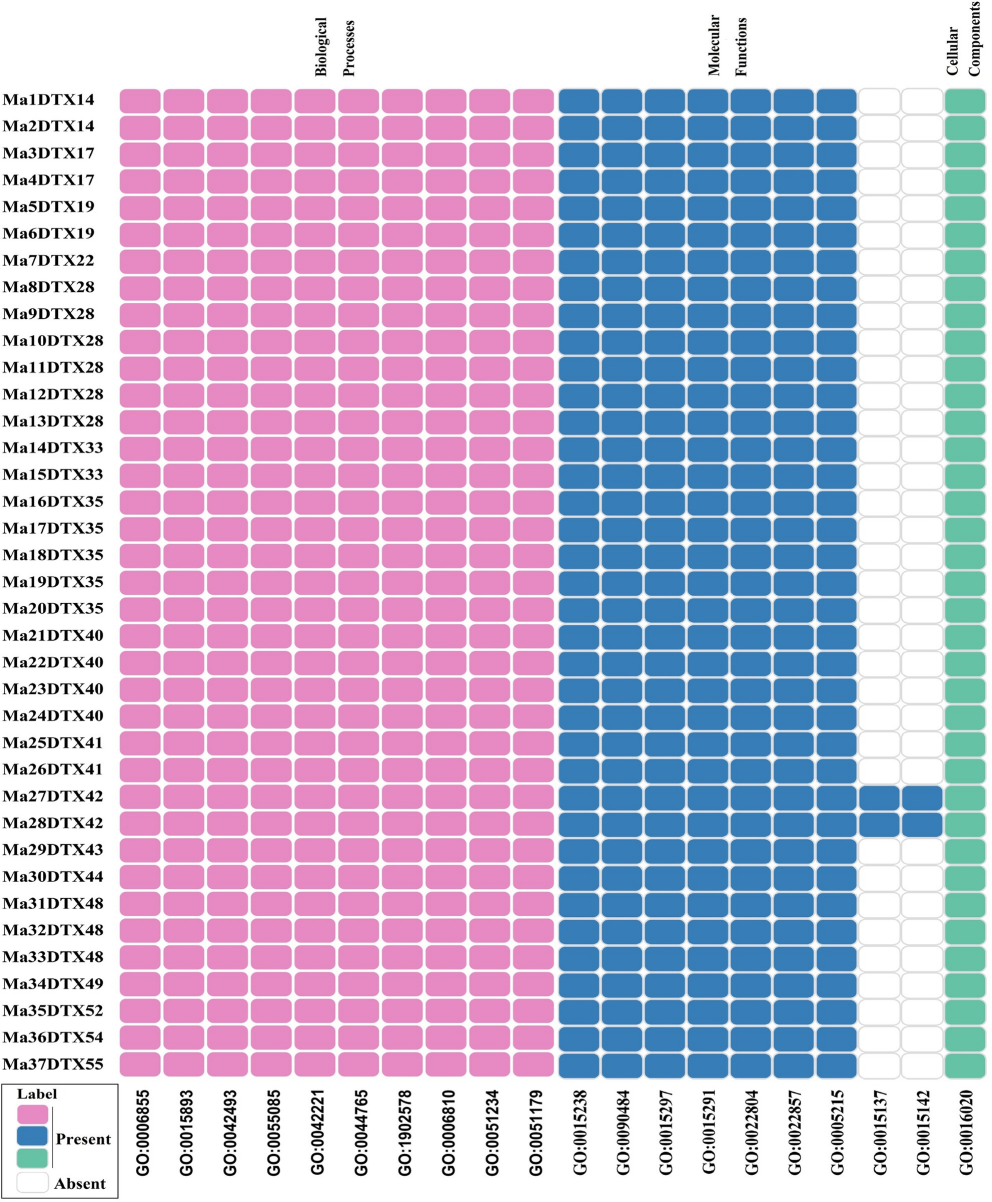
The heatmap for the predicted GO terms corresponding to the predicted MaDTX genes in banana. The pink, blue and sky blue color represent presence and white color indicate absent of the genes.

The MaDTX genes encoding DTX proteins were analyzed using the GO database, PlantTFDB in order to predict the location or functional similarity and to understand the biological roles of genes. Genes associated with different gene ontological terms describe the involvement of genes with various functional pathways. The GO analysis revealed that 37 MaDTX genes were involved in gene ontology annotations categorized under cellular structural component (CC), molecular functions (MF), and biological processes (BP) (Fig 7). MaDTX genes were identified to participate in the cellular structural component aspect pertaining to membranes. Regarding molecular functions (MF), the predicted genes were involved in various types of transporter activities. In terms of biological processes (BP), the predicted banana DTX genes were associated with functionalities related to reacting to drug and chemical stimuli, processes of localization, and the transportation of drugs. In all gene ontology function annotations, genes were involved in different functions which were observed with different GO terms indicate that they play various types of roles. Within the realm of biological processes (BP), the ensuing functional annotations were discerned across all DTX genes in banana: Drug transmembrane transport (GO:0006855), drug transport (GO:0015893), response to drug (GO:0042493), transmembrane transport (GO:0055085), response to chemical (GO:0042221), single-organism transport (GO:0044765), single-organism localization (GO:1902578), transport (GO:0006810), establishment of localization (GO:0051234), and localization (GO:0051179). The following GO functional annotations were found in all MaDTX genes relation to the MF: drug transmembrane transporter activity (GO:0015238), drug transporter activity (GO:0090484), antiporter activity (GO:0015297), secondary active transmembrane transporter activity (GO:0015291), active transmembrane transporter activity (GO:0022804), transmembrane transporter activity (GO:0022857), and transporter activity (GO:0005215). In addition, citrate transmembrane transporter activity (GO: 0014137), tricarboxylic acid transmembrane transporter activity (GO:0015142) were found only in Ma27DTX42, Ma28DTX42 genes. In CC, membrane (GO:0016020), was found in GO functional annotation.

### 3.3 MaDTXs gene regulatory elements analyses

#### 3.3.1. Exploring key transcription factors (TFs) as the regulators of MaDTXs

**Fig 8:**
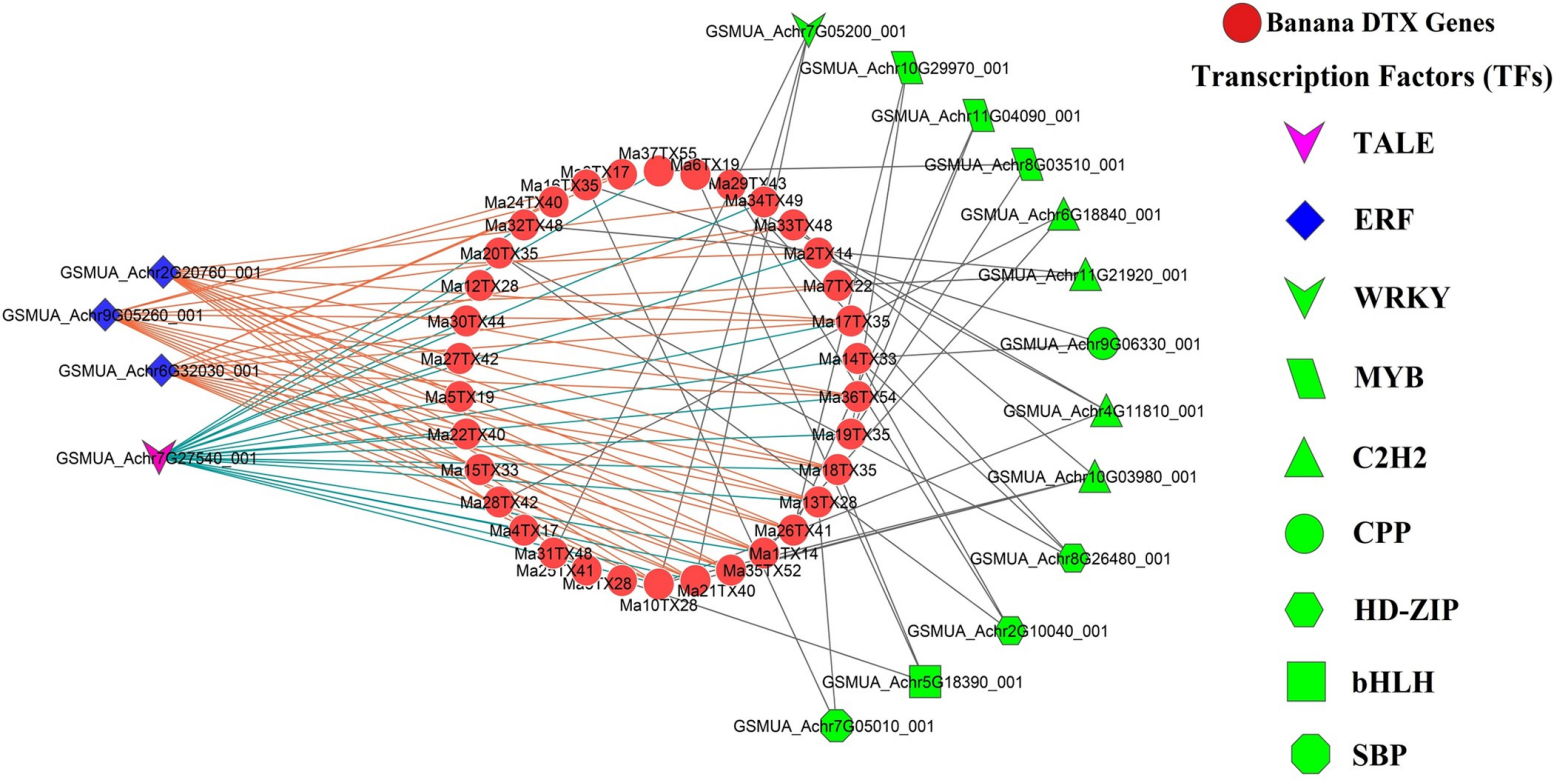
The regulatory network among the TFs and MaDTX proteins. The nodes of the network were colored based on DTX genes and TFs. DTX genes were represented by orange color and the TALE, ERF and pink, blue and green color stand in for other TFs.

Transcription factors (TFs) serve as crucial regulators of gene expression in living organisms by modulating the initiation and rate of genetic transcription, thereby coordinating the activities within genetic networks [51, 52]. A multitude of transcription factor (TF) families are linked to diverse aspects of plant biology, including growth regulation, developmental processes, responses to both abiotic and biotic stresses, metabolic pathways, and fortification against microbial infections [51, 53]. Thus, identifying of the regulatory transcription factors (TFs) of the predicted DTXs genes can help to develop understanding of gene expression process in banana. In this study, a total of 92 transcription factors (TFs) were identified who regulate the anticipated DTX genes in entire banana genome (Fig 8). The identified TFs were divided into 9 TF families: TALE, ERF, WRKY, MYB, C2H2, CPP, HD-ZIP, bHLH, and SBP. Among the TFs families, TALE and ERF are the top two, containing 20 and 39 transcription factors respectively, accounting for 64.13% of the total identified TFs. According to the network analysis, the identified TFs family displayed a distinct structure that linked to the predicted banana DTX genes. The following genes are regulated by ERF Transcription factor family: Ma35TX52, Ma13TX28, Ma3TX17, Ma36TX54, Ma5TX19, Ma21TX40, Ma1TX14, Ma25TX41, Ma34TX49, Ma18TX35, Ma10TX28, Ma7TX22, Ma26TX41, Ma24TX40, Ma17TX35 and Ma33TX48. On the other hand, Ma35TX52, Ma13TX28, Ma36TX54, Ma4TX17, Ma30TX44, Ma14TX33, Ma21TX40, Ma37TX55, Ma1TX14, Ma27TX42, Ma2TX14, Ma15TX33, Ma19TX35, Ma34TX49, Ma22TX40, Ma32TX48, Ma18TX35, Ma10TX28, Ma12TX28, Ma17TX35 genes are associated with the TALE transcription factor family.

#### 3.3.2. Exploring key microRNAs as the post-transcriptional regulators of MaDTXs

**Fig 9:**
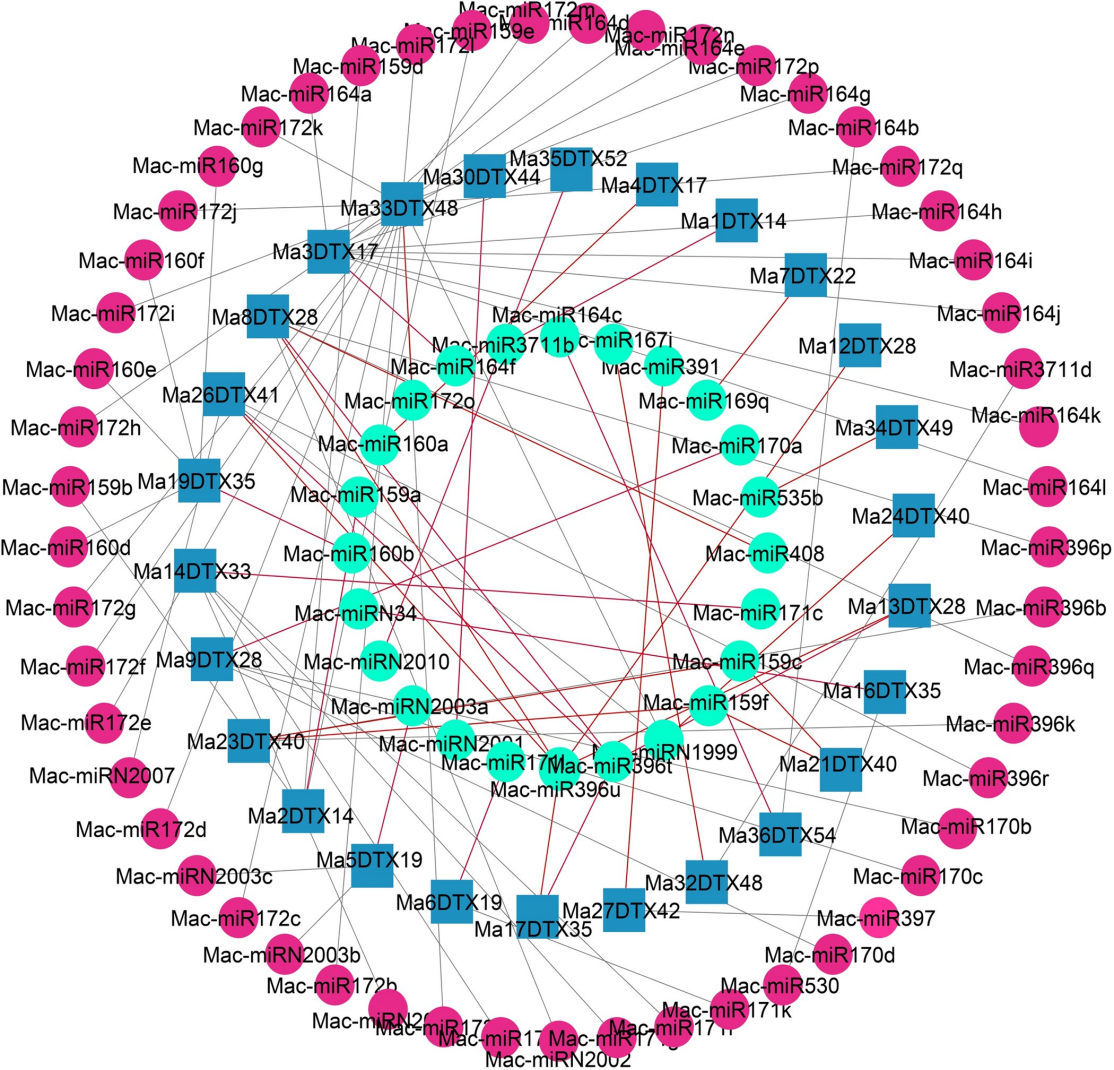
The regulatory network among the predicted miRNA and MaDTX genes. The square and round boxes represent the targeted MaDTX genes and miRNA. The cyan circle represent the miRNAs related to growth, development and protection of banana plant.

MicroRNAs (miRNAs) are essential regulators of gene expression in plants. The retrieval of miRNA sequences was conducted utilizing the Plant MicroRNA Encyclopedia database. The psRNATarget online tool was used to determine the miRNAs that could potentially target banana (*M*. *acuminata*) DTX genes. The investigation revealed that a collective sum of 88 miRNAs were identified to specifically target 26 out of the 37 genes belonging to the DTX family in banana (Fig 9). None of the remaining 11 DTX genes were targeted by these miRNAs. The length of these miRNAs ranged from 20 to 24 amino acids. The number of miRNAs that targeted these genes ranged from 1 to 18. The banana Ma1DTX14, Ma4DTX17, Ma7DTX22, Ma12DTX28, Ma24DTX40, Ma30DTX44, Ma34DTX49 and Ma35DTX52 genes were targeted by only 1 mature miRNA. On the other hand, Ma6DTX19, Ma13DTX28, Ma16DTX35, Ma17DTX35, Ma21DTX40, Ma27DTX42, Ma32DTX48 and Ma36DTX54 genes were targeted by 2 miRNA. The Ma5DTX19, Ma8DTX28 and Ma3DTX17 genes were targeted by 3, 6 and 10 mature miRNAs respectively. The Ma2DTX14, Ma9DTX28, Ma23DTX40 genes were targeted by 4 miRNA and the Ma14DTX33, Ma19DTX35, Ma26DTX41 genes were targeted by 5 miRNA. The 18 miRNAs were identified to target the Ma33DTX48 gene, making it the sole gene subjected to the highest number of miRNA interactions.

#### 3.3.3. Exploring *cis*-acting regulatory elements of MaDTXs

**Fig 10:**
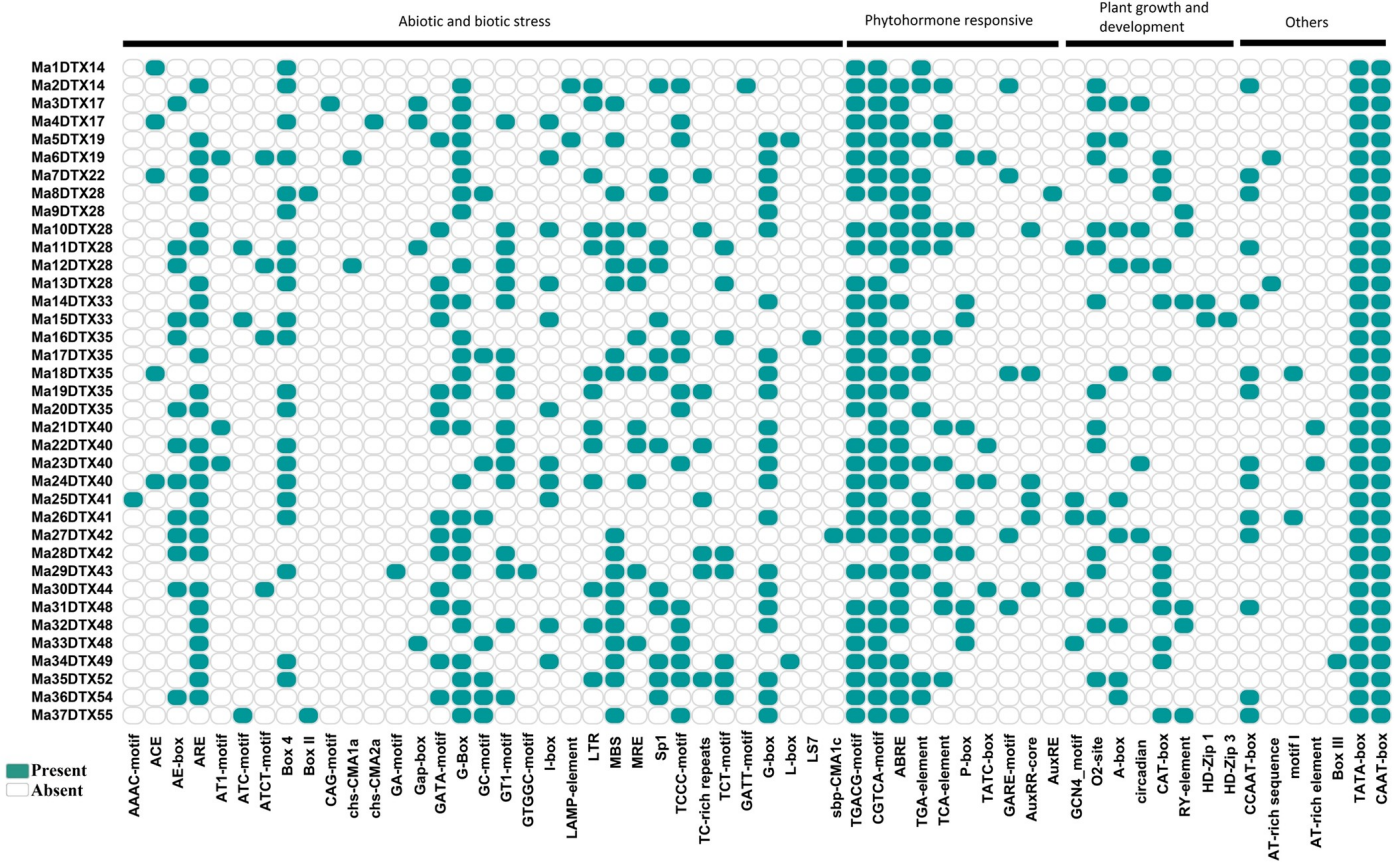
The *cis*-regulatory elements in 1.5 kb upstream region of the identified MaDTX genes. The deep color with circular rectangle shapes represent the presence of that elements alongside their respective genes.

Plant gene transcription is also regulated by *cis*-acting regulatory elements [54]. The regulatory elements that bind to transcription factors in the 1.5 kb promoter region upstream of the gene were identified using the PlantCARE database. The analysis revealed that the *cis*-acting elements segregate into categories such as responses to abiotic and biotic stresses, reactions to phytohormones, involvement in plant growth and development, and other related activities (Fig 10). Large numbers of stress responsive elements such as anaerobic induction (ARE), drought responsive elements (MBS), low-temperature-responsive (LTR) elements, and light responsive elements (G-box), were observed. The phytohormone responsive group had 10 elements, including the MeJA responsive element (TGACG-motif and CGTCA-motif), abscisic acid-responsive elements (ABRE), auxin-responsive elements (TGA-element), which were broadly distributed among the MaDTX genes. Nine *cis*-elements associated with plant growth and development were identified within the promoter regions of MaDTX genes. Among these, the regulation of metabolism (O2-site), expression in meristems (CAT-box), and differentiation of palisade mesophyll cells (HD-Zip 1) were considered particularly significant. TATA and CAAT boxes, the primary *cis*-acting elements in eukaryotes, were present in all the predicted MaDTX promoters.

### 3.4 Expression analysis of MaDTX genes under the biotic and abiotic stresses

**Fig 11:**
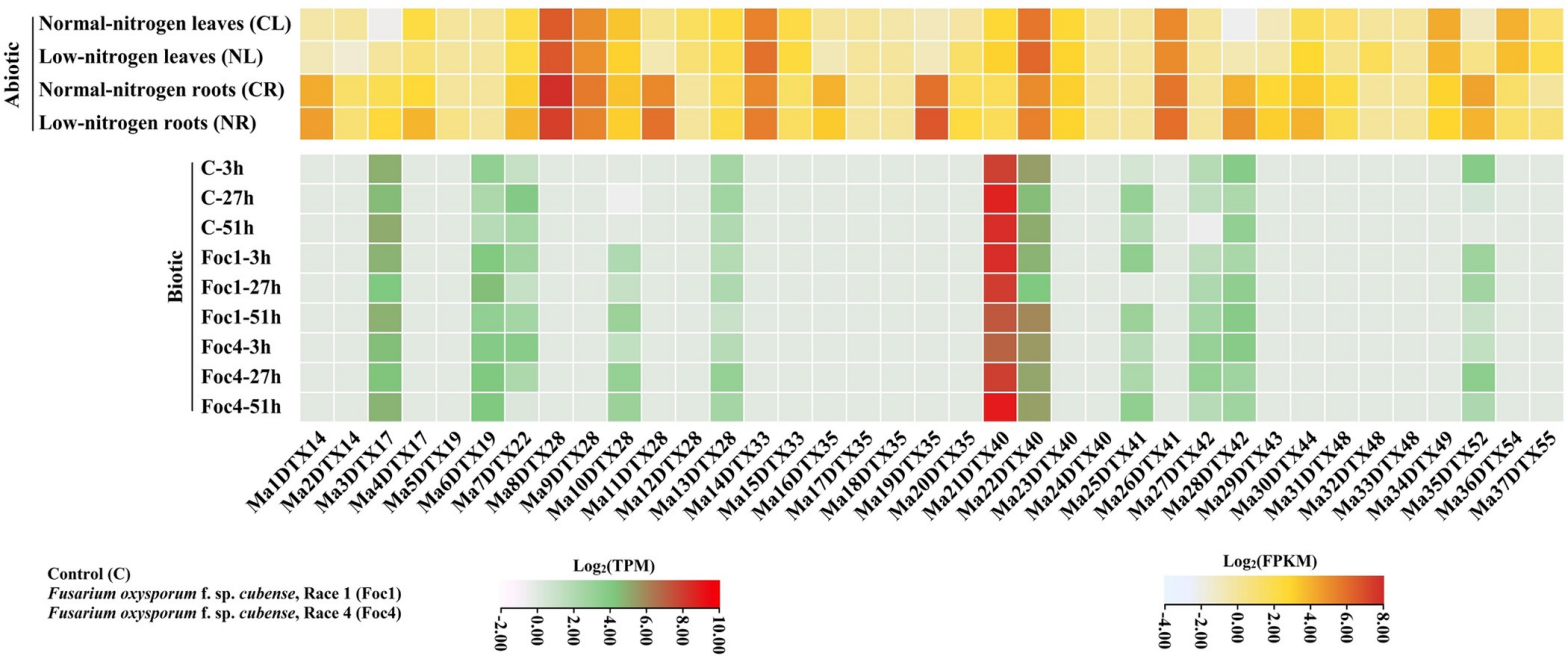
The heatmap of the MaDTX genes in response to *F*. *oxysporum* (race 1 & 4) and low-nitrogen stress. TPM and FPKM values were transformed by log2.

There is a growing body of evidence indicating that DTX genes play a crucial role in providing tolerance to both abiotic and biotic stresses [12, 55–57]. To investigate how MaDTX genes respond to abiotic and biotic stress, the transcriptional changes of MaDTXs were examined when exposed to *Fusarium oxysporum* f. sp. *cubense* (Foc) and low-nitrogen conditions (Fig 11). After low nitrogen stress, a total of 16 and 14 genes were upregulated and downregulated, respectively in the leaves while a total of 19 and 10 genes were upregulated and down-regulated, respectively, in the roots. Among the top expressed genes, the expression levels of Ma8DTX28 showed up-regulated in the leaves and down-regulated in the roots. The Ma22DTX40 and Ma14DTX33 showed up-regulated both in the leaves and roots. In banana, the quality and yield can be threatened by a soil-borne fungus, *Fusarium oxysporum* f. sp. *cubense* (Foc) [57]. A total of 11 MaDTX genes expressed upon the two race (1&4) of the fungus. The Ma21DTX40 gene exhibited higher expression at 27h and 51h in response to race 1, but showed down-regulation at 3h and 27h in response to race 4, whereas it showed up-regulation at 3h by race 1 and 51h by race 4. These findings suggest that the DTX gene family in bananas plays a varied role in responding to both biotic and abiotic stresses, particularly in the context of *F*. *oxysporum* infection and low-nitrogen stress.

## 4. Discussion

Plants employ various defense mechanisms to protect themselves from adverse environmental conditions. One such mechanism involves removing harmful substances from cells using transporters called multidrug efflux pumps among which the multidrug and toxic compound extrusion family (MATE)/detoxification efflux carrier (DTX) is particularly significant [58]. This study conducted a comprehensive computational analysis of DTX gene family in banana (*M*. *acuminata*) and identified 37 DTXs genes in the entire banana genome. A phylogenetic tree analysis was performed utilizing the newly detected 37 MaDTXs protein sequences with 56 AtDTXs to investigate the evolutionary relationship among the DTX proteins. The MaDTX transporters were then divided into four groups based on the classification of AtDTX proteins. Group I consists of 6 MaDTX transporters and 19 AtDTX proteins (AtDTX1 to AtDTX19). Within this group, the proteins AtDTX1 and AtDTX4 are capable of facilitating the removal of alkaloids or external harmful substances from the cell’s cytoplasm, and they also contribute to auxin synthesis in *Arabidopsis* [11]. The AtDTX14 and AtDTX18 are responsible for excluding norfloxacin and secret coumaroylagmatine and other hydroxycinnamic acid to protect the plant from *P*. *infestans* [16, 59]. The aberrant lateral root formation 5 (ALF5/AtDTX19) gene exhibits expression within the root epidermal cells, facilitating the transportation of tetramethyl ammonium, and plays an essential role in shielding roots against harmful substances present in the soil [13]. Therefore, MaDTX proteins that belonging to group-I may exibit similar functions to AtDTX proteins in this group. The group II contains 20 MaDTX proteins that cluster with 22 AtDTX (AtDTX20-AtDTX41) proteins. Among them, the AtDTX21 and AtDTX41 are involved in atrazine detoxification and transportation of proanthocyanidins or anthocyanins to the vacuole [60, 61]. The AtDTX30, AtDTX31, AtDTX33 and AtDTX35 are associated with root hair development [62, 63]. The AtDTX41/Transparent Testa 12 (TT12) controls the anthocyanin transport *in-vitro* [61]. Thus, the MaDTX proteins belonging to group-II might be responsible for transportation of different phytocompounds and development of the root hair in banana. The group-III comprises of 4 MaDTXs along with 6 AtDTXs (AtDTX42-AtDTX47) proteins. In the presence of biotic stress, the AtDTX47 protein contributes to the plant’s immune response by expelling salicylic acid (SA) [64]. Furthermore, the essential role of ferric reductase defective 3 (FRD3/AtDTX43) lies in ensuring zinc tolerance through the regulation of iron balance [65]. Therefore, the MaDTX proteins in group-III could potentially play a role in plant tolerance and defense against both biological and environmental stresses. Group IV contains 7 MaDTX transporters along with 9 AtDTXs (AtDTX48-AtDTX56). The bush and chlorotic dwarf 1 (AtDTX48) gene exhibit a spectrum of roles encompassing organogenesis, iron equilibrium maintenance, and elongation of hypocotyl cells, actively expressed specifically within floral structures [66, 67]. The AtDTX50 is capable of transporting abscisic acid (ABA) and responding to drought conditions [68]. The AtDTX51 and AtDTX52 regulate iron homeostasis [69, 70]. The initiation of lateral organ is controlled by AtDTX54 and AtDTX55 [71]. The CO_2_-induced signaling of stomatal closing in plant leaves is regulated by the AtDTX56 transporter [72]. Thus, the MaDTX proteins in group-IV may be engaged in iron homeostasis and transportation different molecules involved in the development, and growth of banana. However, AtDTX transporters have so many other diverse functions [13, 60, 62, 63, 66, 73], which indicate that MaDTX proteins may likewise be potentially responsible for performing other functions.

In the motif analysis, all motifs selected among different DTX family genes of banana and *Arabidopsis* were almost similar, corresponding to the similarities in phylogenetic trees (Fig 4). The motifs of groups I, II and IV share a similar pattern and are distinctly different from group III, which is same as DTX genes in soybean and flax [22, 74]. The structures of MaDTX family genes are almost similar to the structures of AtDTX family genes in the same group but different from the genes belonging to other groups (Fig 5). Each of MaDTX genes contains at least one intron which might be played a vital role for recombination in the construction of new combinations of exons [75]. The expression of the DTX genes might depend on the loss or gain of introns, similar to cotton, maize and flax [20, 74, 76]. Chromosomal location analysis of MaDTXs reveals that most of the MaDTX genes are positioned on the arms of chromosomes, which are related to substantial rates of recombination [77]. The localization of most of the MaDTX proteins within the plasma membrane (Fig 6) clarifies that their function is to maintain the integrity of plasma membrane by extruding different drug molecules from the plants [78].

The Gene Ontology (GO) annotation anticipates the possible functions of MaDTX genes might have within the cells that are over- or under-expressed (Fig 7) [79]. Cellular detoxification processes involve transporting xenobiotic (GO:0006855) across a membrane [80]. Another mechanism called response to drug (GO: 0042493), and response to different chemicals (GO:0042221), results in changes in various functions like movement, secretion and enzyme production [81]. The movement of different substances such as, macromolecules, micromolecules, ions, and cellular organelles into or out of a cell or between cells, is maintained by transporters in the plasma membrane (GO:0006810, GO:0044765) [82]. The predicted MaDTXs gene products involved in different biochemical activities, including transportation of drugs, citrate, and tricarboxylic acid from one side to another side of a membrane (GO:0015238, GO:0090484, GO:0015137, GO:0015142), and transfer of solute across membrane using a chemiosmotic energy source (GO:0015291) [83–85]. Among the three categories of gene ontology, the cellular component reveals that all the predicted MaDTXs gene products are present in the lipid bilayer membrane of a cell or organelles, including nucleus (GO:0016021) [86]. Thus, the outcomes of the GO enrichment analysis indicated that the envisaged MaDTX genes potentially exert a crucial function in safeguarding cellular integrity and membrane-bound organelles through detoxification mechanisms, eliminating pharmaceutical compounds from the cells of the banana.

Different transcription factors (TFs) have been found to be important regulators of developmental processes, stress responses, and genetic control in plants [87]. In this investigation, 9 TF families (TALE, ERF, WRKY, MYB, C2H2, CPP, HD-ZIP, bHLH and SBP) comprising 92 transcription factors (TFs) were identified, where TALE and ERF are the key transcriptional regulators of MaDTX genes (Fig 8). The three-amino-acid-loop-extension (TALE) transcription factor, a class of homeoproteins, is responsible for controlling the development and/or maintenance of shoot apical meristem, plant architecture, and many features of the reproductive stages [88]. The Ethylene Responsive Factor (ERF) involved in regulation of genes in plants associated with various biological processes such as, plant growth and development, stress responses, using transcriptional and post-transcriptional control mechanism [89, 90]. The myeloblastosis (MYB) proteins are found in both plants and animals which, playing profound roles in metabolism and stress responses in *Arabidopsis* [91, 92]. Moreover, the WRKY, C2H2 and CPP transcription factors are also involved in stress responses [93–95]. Together with the findings of this investigation, these studies strongly support the role of MaDTX genes in growth and survival under various stress conditions.

MicroRNAs (miRNAs) are endogenous regulators composed of 20 to 24 nucleotide in plant that regulate different kinds of vital biological processes, including growth and development, dealing with pathogens, and maintaining appropriate internal conditions in plants [47, 96]. They regulate gene expression post-transcriptionally by repressing of protein production and inducing translational gene silencing [97]. A collective of 88 microRNAs was identified, directing their action towards 26 out of the 37 DTX family genes in banana (Fig 9). The miR159 family, widely prevalent across the plant realm, plays a pivotal role in transitioning plants from vegetative to reproductive phases and governing the development of flowers [98]. The miR156 group targeted 3 MaDTX genes, namely, Ma2DTX14, Ma21DTX40 and Ma23DTX40. This might suggest that these three genes might be associated with enhancing vegetative growth and suppressing reproductive development in banana. The Ma3DTX17 and Ma36DTX54 genes in banana were found to be targeted by the miR164 group that is involved development vegetative and reproductive part of *Arabidopsis* and tomato [99]. Several studies found that the miR172 family involved in different types of biological processes at post-transcriptional level including transition from immature to adult stage, vegetative to reproductive phase, and floral development [100–103]. In banana, this miR172 was observed targeting the Ma33DTX48 gene. Hence, this gene could potentially exhibit analogous functionalities to any or all of the specified roles attributed to miR172. The miR396 molecules participate in the modulation of abiotic stress reactions in *Arabidopsis* and pitaya [104]. In banana, this miR396 was observed to target six genes, namely, Ma8DTX28, Ma12DTX28, Ma13DTX28, Ma17DTX35, Ma23DTX40, Ma26DTX41, and it can be inferred that these MaDTX genes protect banana plant from adverse environmental conditions.

The regulatory elements present in the promoter region exert a pivotal function in controlling how genes are transcribed in response to different environmental cues, such as biotic and abiotic factors, hormones, and in overseeing developmental processes in plants [105]. The abscisic acid responsive element (ABRE) is important in response to dehydration and salinity in plants [106]. The O_2_-site *cis*-acting element is related to zein metabolism regulation [107]. The TCA-element and TC-rich repeats in promoter region withstand environmental stresses including biotic and abiotic stresses [108–110]. The ARE motif regulate gene expression with low oxygen level in plants [111]. The G-box (CACGTG) *cis*-element plays a significant role in response to light, methyl-jasmonate, and involved in ethylene induction in plant [112]. The MBS *cis*-element binds to MYB transcriptional factors, which regulate the drought stress signaling pathway [113]. Conversely, the LTR motif is responsible for the activation of cold-regulated genes [114]. Another well-studied *cis*-element found in plants is the TGACG and CGTCA motif, which are methyl jasmonate responsive elements in plant [115, 116]. Overall, *cis*-elements analysis further confirmed that the MaDTX genes in banana have a substantial impact on how plants grow and develop in response to various biotic and abiotic stresses.

By analyzing changes in the expression levels of MaDTX genes before and after fungal infection and low-nitrogen stress that have a great impact on the quality and yield of banana and development of plant, suggesting that MaDTX genes are involved in banana response to infection, especially for *F*. *oxysporum* and low-nitrogen stress (Fig 11). Notably, Ma22DTX40 is involved in both fungal infection and low-nitrogen stress. Thus, we speculated that the sustained expression of Ma22DTX40 is important for apple growth and stress response. Additionally, the promoter regions of all MaDTX genes contain many stress response *cis*-elements and we further speculated that the other genes, which are not involved in the fungal infections and low-nitrogen stress has great potential for stress response.

## 5. Conclusion

In this study, integrated bioinformatics methods were employed to identify potential DTX family genes in the banana (*M*. *acuminata*) genome. Our analysis revealed the presence of 37 MaDTX genes, categorized into four distinct groups based on phylogenetic analysis. Further analysis of conserved domains, motifs, and gene structures suggested that MaDTX genes may function similarly to AtDTX genes as indicated by their phylogenetic relationship. Sub-cellular localization prediction showed that most MaDTX proteins are predominantly located in the plasma membrane. Gene Ontology (GO) analysis indicated that the majority of predicted MaDTX genes are associated with important biological processes, molecular functions and cellular components. Furthermore, our identification of regulators of MaDTX genes, including transcription factors (TFs), microRNAs (miRNAs), and *cis*-acting elements, suggests their involvement in environmental stress responses, as well as the growth and development of bananas. Notably, changes in gene expression levels in response to pathogenic infections and non-living factor indicate that MaDTX genes play a role in responses to both biotic and abiotic stresses. Overall, our findings provide a foundation for future experimental investigations in the laboratory to elucidate the precise roles of MaDTX family genes in disease resilience, stress responses, and the growth and developmental processes specific to bananas.

## Supporting information

S1 Fig The phylogenetic tree of banana, *Arabidopsis* and rice DTX proteins. (TIF)

S2 Fig Chromosome distribution of the predicted MaDTX genes in *M*. *acuminata*. (TIF)

S1 File Full-length protein sequences of DTX gene families of *A*. *thaliana*, *M*. *acuminata* and *O*. *sativa*. (TXT)

S2 File The details GO analysis of the predicted DTX family genes. (XLSX)

S3 File Identified 92 TFs associated the regulation of predicted DTX family genes in banana genome. (XLSX)

S4 File The anticipated 88 miRNAs that targeted 26 out of 37 DTX family genes in banana genome. (XLSX)

S5 File The predicted *cis*-acting regulatory elements of DTX gene family in banana. (XLSX)

S6 File TPM and FPKM values of MaDTX genes with *F*. *oxysporum* and nitrogen stresses. (XLSX)
