# Supplementary figures and images for: Genome-wide identification and characterization of DTX family genes highlighting their locations, functions, and regulatory factors in banana (Musa acuminata)
Source: PLoS One. 2024 Jun 6;19(6):e0303065. doi: 10.1371/journal.pone.0303065 (PMC11156367; doi:10.1371/journal.pone.0303065)

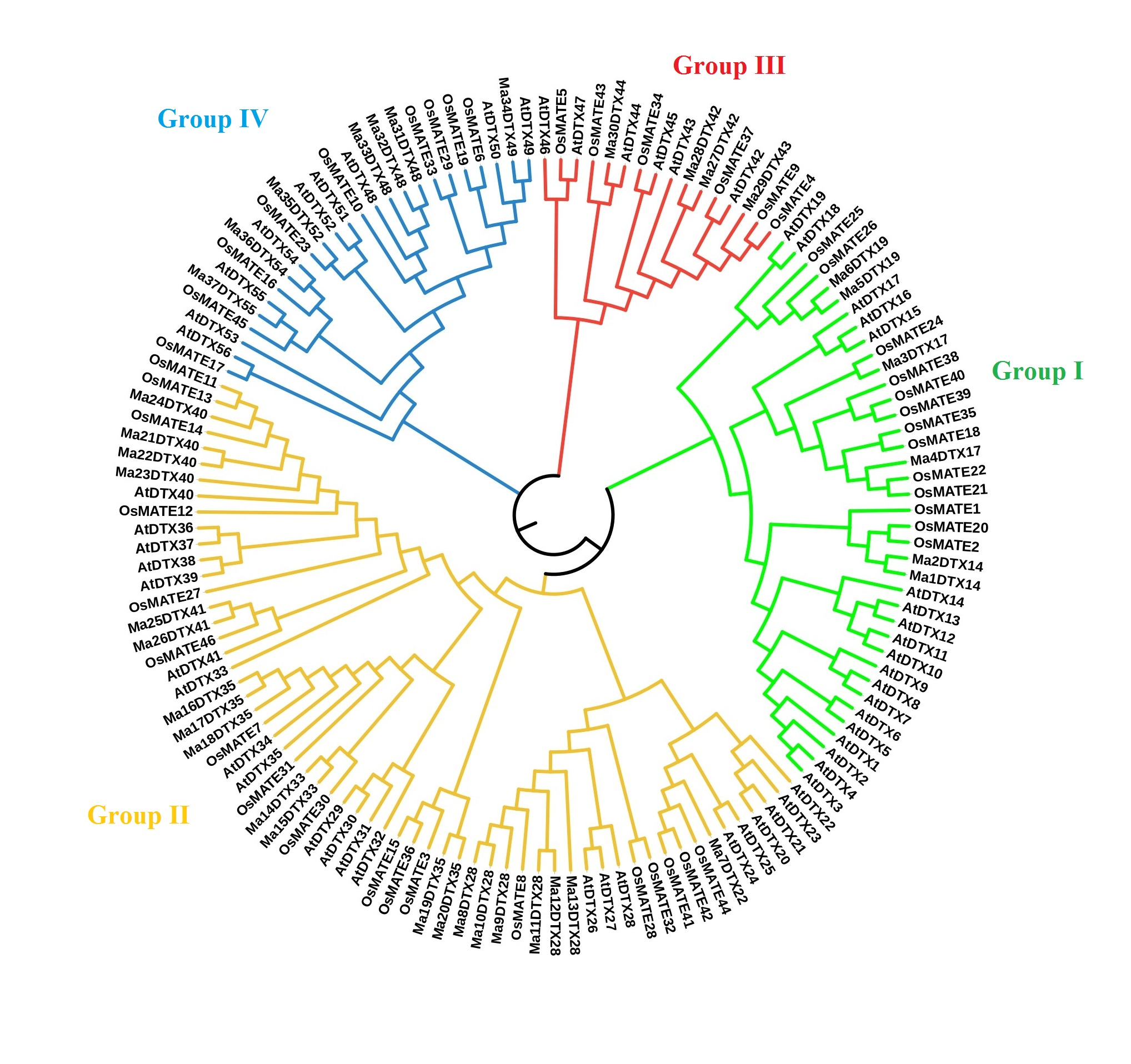

Supplement: S1 Fig — (TIF) [file pone.0303065.s001.tif]

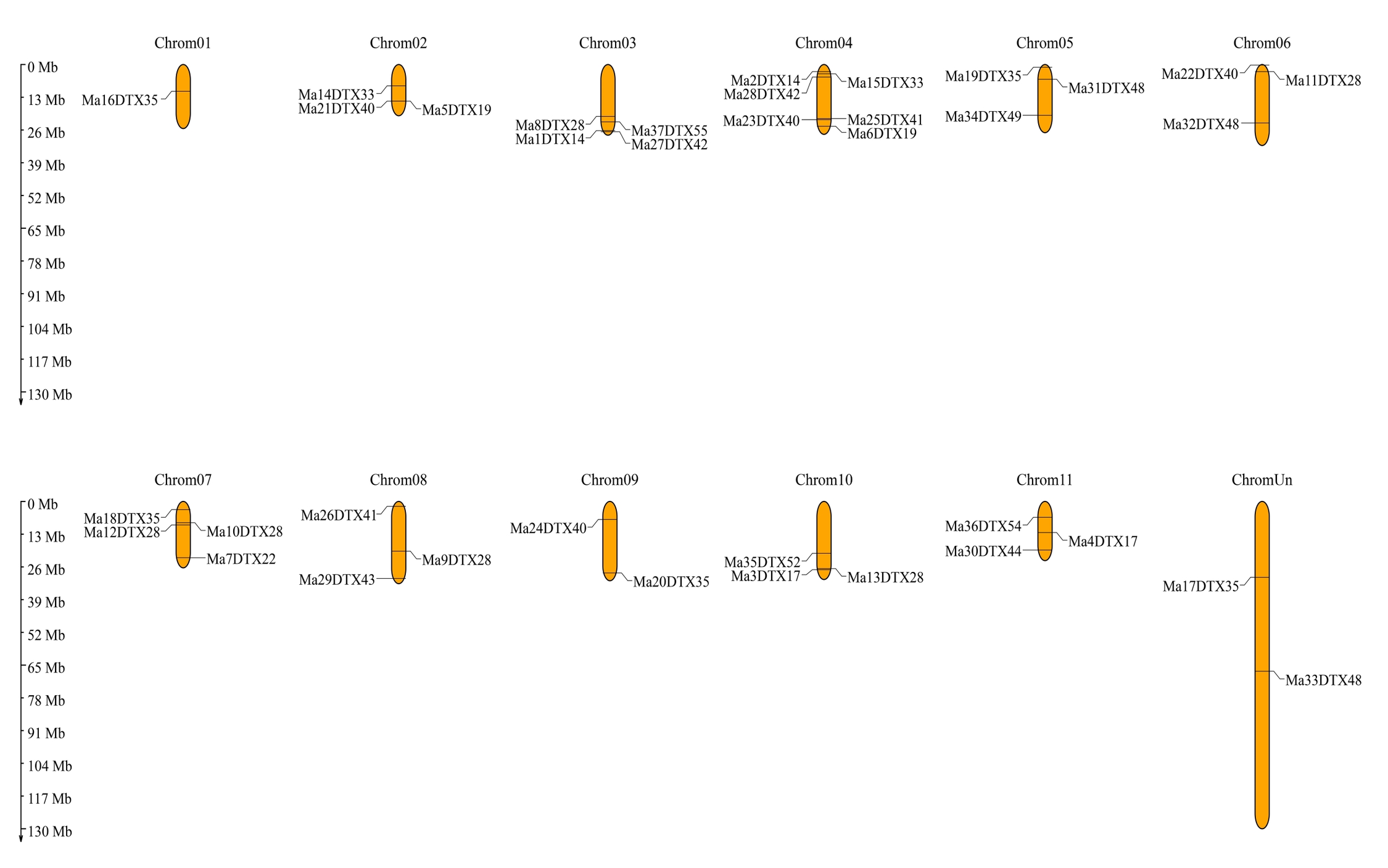

Supplement: S2 Fig — (TIF) [file pone.0303065.s002.tif]
